# Validation of Vibration Exercises on Enhancing Muscle Strength and Upper Limb Functionality among Pre-Frail Community-Dwelling Older Adults

**DOI:** 10.3390/ijerph192114509

**Published:** 2022-11-04

**Authors:** Chia-Hui Kao, Shang-Lin Chiang, Li-Wei Chou, Chia-Huei Lin, Yueh-Hsun Lu, Liang-Hsuan Lu, Xin-Miao Wang, Chueh-Ho Lin

**Affiliations:** 1 Master Program in Long-Term Care, College of Nursing, Taipei Medical University, Taipei 110, Taiwan; 2 Department of Physical Medicine and Rehabilitation, Tri-Service General Hospital, School of Medicine, National Defense Medical Center, Taipei 114, Taiwan; 3 Department of Physical Therapy and Assistive Technology, National Yang Ming Chiao Tung University, Taipei 112, Taiwan; 4 Department of Nursing, Tri-Service General Hospital, School of Nursing, National Defense Medical Center, Taipei 114, Taiwan; 5 Department of Radiology, Shuang-Ho Hospital, Taipei Medical University, Taipei 110, Taiwan; 6 Department of Radiology, School of Medicine, College of Medicine, Taipei Medical University, Taipei 110, Taiwan; 7 Taipei Neuroscience Institute, Taipei Medical University, Taipei 110, Taiwan; 8 Faculty of Humanities, Zhejiang Dong Fang Polytechnic College, Wenzhou 325011, China; 9 International Ph.D. Program in Gerontology and Long-Term Care, College of Nursing, Taipei Medical University, Taipei 110, Taiwan; 10 Center for Nursing and Healthcare Research in Clinical Practice Application, Wan Fang Hospital, Taipei Medical University, Taipei 110, Taiwan

**Keywords:** frailty, rehabilitation, vibration exercises, Taiwan, gerontology

## Abstract

Pre-frail older adults require appropriate exercise to enhance muscle strength as well as upper limb functionality. We developed a handheld vibrator and evaluated its effectiveness in enhancing muscle strength compared to conventional exercises among pre-frail community-dwelling older adults. Thirty-one pre-frail older adults (aged 75.5 ± 5.1 years) were recruited and randomly allocated to a vibration group (VG) and control group (CG). The VG underwent 20 minutes of vibration (frequency: 30 Hz, amplitude: 5 mm, horizontal vibration) using a handheld vibrator as well as 40 minutes of conventional exercise. The CG received 60 minutes of conventional exercise only. The primary outcome was muscle strength assessment (kg), and the secondary outcome included activities of daily living and instrumental activities of daily living scores. The comparisons between the outcome measures revealed no significant differences at the baseline level. Muscle strength of the dominant (*ß* = 2.49, *p* = 0.002) and non-dominant (*ß* = 1.89, *p* = 0.02) wrist flexion, brachioradialis (*ß* = 3.8, *p* = 0.01), and biceps brachii (*ß* = 3.02, *p* = 0.02) in the dominant upper limbs was significantly increased among the VG. The vibration intervention can enhance muscle strength in the upper limbs among pre-frail older adults.

## 1. Introduction

Daily activities such as eating, dressing, and writing require muscle strength in the upper extremities. Aging is associated with decreased muscle strength and functional limitations [1]. A study has indicated that aging-induced neuromuscular physiological deterioration could result in motor and functional impairment as well as long-term disabilities among older adults [2]. Studies have also found that decreased muscle strength, muscle atrophy, and decreased functionality are associated with frailty among older adults [1,3]. These aspects may result in increased daily living dependency levels and poorer health conditions among frail older adults. The prevalence of pre-frailty among community-dwelling older adults in low-income and middle-income countries is between 13.4% and 71.6% [4].

Among older adults, early exercise-based interventions aimed at maintaining or enhancing muscle strength and functional performance in their upper extremities during the pre-frailty stage are required to avoid further frailty, functional limitations, and poor health conditions. Chen et al. reported that elastic band-based exercises enhance muscle strength, increase physical activity, and reduce frailty among pre-frail older adults [5]. Owing to the high prevalence of hypertension (70%) among frail older individuals, healthcare professionals should be wary of sudden and unstable cardiovascular loads during physical activity [6]. Exercise-based interventions aimed at enhancing muscle strength may induce cardiovascular responses, such as increased heart rate, blood pressure, and oxygen consumption, which could result in discomfort among older adults, thereby requiring significant caution during the implementation process [7]. Contrarily, recent studies on this subject have suggested that vibration exercises are brief and safe training- and exercise-based interventions. Vibration exercises do not induce significant increases in heart rate, blood pressure, or oxygen consumption [8]. Such exercise-based interventions may enhance muscle strength and functionality in the lower extremities [9] without inducing negative factors, such as hot feet, itching, pain, vertigo, and hip discomfort [10].

Vibration exercises induce enhanced α-motor neuron excitability, motor unit recruitment, and discharge rate via the central and spinal neural mechanisms, such as the tonic vibration reflex, thereby resulting in increased electromyography scores and muscle force [10,11]. Stania et al. (2016) reported that in many studies, researchers used vibration exercises to enhance muscle strength and lower limb functionality among older adults. However, few studies focus on the upper limbs because of the lack of appropriate vibration devices specifically aimed at enhancing upper-limb muscle activity and functionality. For example, in recent studies, researchers used vibratory platforms, whereby participants were asked to take specific positions (sit on a chair or kneel on the ground and then support their upper limbs over the vibratory platform with their hands or elbows shoulder-width apart) for exercises aimed at enhancing upper-limb muscle activation [12,13,14,15,16,17,18,19]. However, such approaches might induce unprecedented upper-limb muscle activation, thereby resulting in injury, dizziness, or discomfort when whole-body vibration is applied [20,21]. Such approaches, as they pertain to vibration exercises, may generate excess vibration forces from the platform and transmit these vibrations to the head [21]. As a result, an appropriate vibrator that is specifically focused on enhancing muscle strength in the upper limbs is required. Therefore, the purpose of this study involves designing a handheld vibrator that focuses specifically on the activation of upper-limb muscles and investigates the effects of vibration exercises on enhancing muscle strength and upper limb functionality among pre-frail older adults.

## 2. Research Design and Methods

### 2.1. Study Design

This was a single-blinded intervention study aimed at evaluating grip strength, upper-limb muscle strength, activities of daily living (ADL), and instrumental activities of daily living (IADL) levels among pre-frail community-dwelling older adults through a randomized trial comparing the impacts of vibration exercises on two groups of the sample population (the vibration group [VG] and the control group [CG]) via a multi-component exercise. To avoid selection bias, the investigator used a generated random number (1 = VG, 2 = CG) for each participant through a randomization approach based on LabVIEW (2015 edition, National Instruments, Austin, TX, USA). In this study, we investigated the impacts of vibration exercises on enhancing muscle strength and upper limb functionality among pre-frail community-dwelling older adults in Taiwan (trial registry no. NCT04767932: retrospectively registered). A blinded statistician analyzed the data associated with muscle strength and upper limb functionality scores among the participants involved in this study.

### 2.2. Participants

Sample size estimation was conducted via G*Power (version 3.1.9, Universität Düs- seldorf, Düsseldor, Germany) software based on repeated analysis of variance (ANOVA) measures (within-between interaction), with a medium-to-small effect size of 0.3, Cronbach’s alpha set at 0.05, a power of 0.80, and two measurements, which resulted in a total of 24 eligible participants. Considering the 20% dropout rate, a minimum sample size of 29 participants was required.

**Figure 1:**
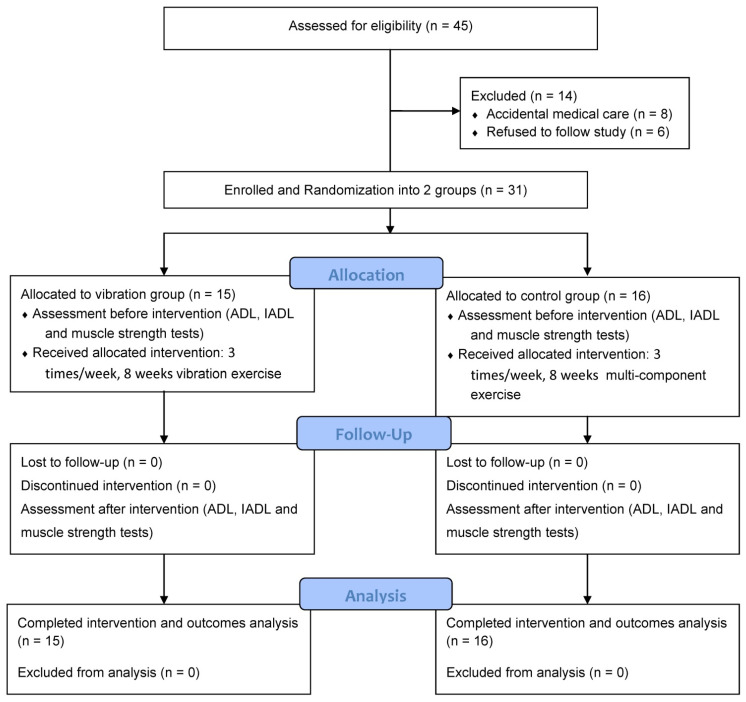
Flow diagram of the randomization procedure and outcome measurements used in the study.

As per the inclusion and exclusion criteria for participation, between August 2020 and March 2021, 45 pre-frail older adults from local communities were invited by a blinded researcher to participate in this study via oral recruitment. The inclusion criteria were as follows: (1) individuals aged between 65 and 85 years, (2) individuals meeting the criteria outlined in the Fried frailty index (FFI), as it pertains to pre-frailty [22], (3) individuals with a Mini-Mental State Examination score of ≥ 21, and (4) individuals that can perform interventions for 60 min without discomfort. The exclusion criteria were as follows: (1) individuals with neurologic or neuromuscular diseases affecting their upper limbs over the past six months, (2) individuals experiencing pain or discomfort (dizziness and vomiting) while performing exercises and evaluation, (3) individuals suffering from acute inflammatory medical conditions limiting their full participation throughout the study, (4) individuals with a pacemaker for major heart diseases, (5) individuals with severe visual or hearing impairments, and (6) individuals that cannot understand and follow study instructions. However, before initiating the study procedure, eight participants required medical care or were admitted to the hospital because of skin allergies, fractures, and falls resulting from sudden accidents, and six stated that they could not follow the protocols for personal reasons. Finally, 31 participants were enrolled and randomly assigned to the VG (*n* = 15) and CG (*n* = 16) by the researcher, and they completed the study process. In our final completed sample size (*n* = 31), there was an estimated power of 0.88. A consort flow diagram of this study’s randomization and outcome measurements is presented in Figure 1. Each participant signed an informed consent form before participation. The Institutional Review Board of Taipei Medical University approved the study protocol (no. N201907023, 08/21/2019).

### 2.3. Research Device

For this study, two handheld vibrators (37 × 8.3 × 10.5 cm; weight: 1 kg for each handheld vibrator) were designed and customized by ACCU BALANCES CORP. (New Taipei City, Taiwan). Two vibration motors (ZYT3424D110; 1.833 V = 1 Hz) were mounted in each handheld vibrator, producing 5 mm amplitude and 0–60 Hz frequency vibrations. The rotary potentiometer on the vibration frequency control box could control the vibration frequencies for both handheld vibrators simultaneously. Moreover, a mechanical frequency pointer was located on the box, and it showed the vibration frequencies of both handheld vibrators in real time throughout the vibration exercise.

### 2.4. Vibration Exercise Program in the VG

Participants in the VG were instructed to sit on a high-fixed, no-arm-support chair with their feet positioned flat on the floor. Both shoulders were positioned sideways, slightly apart (20–30-degree abduction) from the trunk, and the elbow was fixed at 90° flexion as the training position. Thereafter, the researcher instructed the participants to firmly hold the red line on the handheld vibrators with both hands and maintain the training position during vibration. In the vibration intervention, early studies revealed that vibration frequencies of 8–46 Hz could facilitate muscle activation in the upper limbs among healthy participants [23,24,25]. However, a vibration frequency of <20 Hz may result in resonance injury and dizziness [20,21], whereas a vibration frequency of >40 Hz could significantly affect posture control, thereby resulting in muscle fatigue. Moreover, an early study reported that whole-body vertical vibration could not significantly increase muscle activation in the upper limbs [26]. Furthermore, our preliminary findings (unpublished observation) indicated that the horizontal medial and lateral vibration direction could significantly facilitate muscle activation compared to the vertical vibration direction by 6.2–9.7% in the upper limbs. Furthermore, 8–40 Hz vibrations with an amplitude between 2.5 mm and 6 mm could significantly improve 9.8–26% of muscle strength among community-dwelling older adults as well as healthy young participants [25,27].

Early study has reported that multi-component exercises can meet the health promotion needs of older adults [28], and multiple studies combining vibration (15–30 min/section) and multi-component exercises have shown significant improvements in muscle strength and activity [11,29]. In addition, recent studies have revealed that a 10–30 min vibration-based exercise intervention can improve functionality among people with disabilities [19,30]. In this study, we selected a combined vibration and multi-component exercise intervention and demonstrated the improvements in muscle strength for pre-frail participants after eight-week interventions. The vibration protocol (30 Hz frequency, 5 mm amplitude, horizontal vibration direction) for the first 20 min followed by 40 min of multi-component exercises were followed in previous studies, three times per week for eight weeks [18,19,30]. For the 20 min long vibration exercise-based intervention, the vibrations were applied for 60 s, with a one-minute resting interval between vibration sessions [27] to avoid muscle adaptation, fatigue, and the risk of injury [10]. The multi-component exercises were the same as CG but took less time than the CG.

### 2.5. Multi-Component Exercises in the CG

Throughout the study period, participants in the CG received 60 min of multi-component exercises three times per week for eight weeks, as directed by a physiotherapist at the health service center in the community. Multi-component exercises included a 10-min warm-up (slow stepping and static stretching for upper limbs and trunk) and a 10-min cool-down (same as a warm-up) period. The main exercise followed ACSM guidelines and previous studies with 40 min of exercise, which included elastic resistance (elbow flexion, shoulder flexion/extension/abduction/external rotation, 3.7–5.8 pounds, 10 min, 10–15 repetitions), aerobics (quick walking, stepping, and squatting; 20 min, rating perceived exertion scale between 12–14), and balance or agility exercises (tandem and crossover walking, standing on one leg, walking in a figure eight, 10 min) [31,32].

### 2.6. Outcome Measurements

The primary outcome was muscle strength assessment using Jamar and MicoFET3 for bilateral upper limbs. The Jamar hand dynamometer (Lafayette Instrument, Lafayette, IN, USA) has been applied to evaluate the maximal voluntary grip force for both hands with excellent reliability among older [33] participants (intraclass correlation coefficients were 0.82 and 0.97–0.98, respectively). For the grip strength test, the test position for all the participants followed the recommendations of the American Society of Hand Therapists [34]. The participants were asked to hold the Jamar dynamometer (set at the second handle position) with the shoulder adducted and placed in neutral rotation with the elbow joint in 90° flexion, the forearm in a neutral position, and the wrist positioned between 0° and 30° extension, and perform the maximal voluntary grip-force contraction test for three trials [35]. The maximal voluntary contraction value in kilograms was defined as the average maximal voluntary contraction value from the three trials in each hand. For muscle strength assessments, MicoFET3 has demonstrated excellent reliability among older (ICC between 0.90–0.93) [36] participants. In this study, the MicoFET3 was utilized to measure wrist flexor, wrist extensor, and brachioradialis in the forearm, biceps and triceps brachii, anterior lateral and posterior aspect of the deltoid, and supraspinatus in the upper limbs. The testing procedures followed those used in previous studies [37,38]. Secondary outcome evaluation included ADL and IADL scales with strong evidence of reliability and validity [39].

These primary and secondary outcome measurements were evaluated to validate the impacts of vibration exercises on enhancing muscle strength and self-care ADLs among pre-frail older adults before and after the study intervention between both groups. To assist each pre-frail older adult in remembering and completing the entire study process, we assigned a research assistant to monitor and ensure that all participants adhered to the vibration exercise program and multi-component exercises and that pre-and post-outcome assessments and any side effects and regular health education programs were recorded and delivered on time in both groups.

### 2.7. Statistical Analysis

Descriptive statistics, including means and standard deviations (SD), were determined among the participants. The Mann—Whitney U test was used to compare the pre-and post-intervention differences between groups. A Wilcoxon signed-rank test was applied to compare the differences between the pre- and post-tests. The standardized mean difference effect size (d), designed for contrasting two groups on a continuous variable or estimated based on a dichotomous variable, was presented at the pre- and post-intervention differences between groups. Generalized estimating equations (GEEs), which extend the generalized linear model by providing support for non-independent data, such as longitudinal data or repeat measures, were used to estimate the intervention effects of the two groups through a significant interaction of group and time (group × time) [40]. These equations use dependent variables with non-normal distributions and can be applied to evaluate the main effects (group difference: between-group; time effect: within-group) and interactions (intervention effect: group × time) [40]. All statistical analyses were two-tailed and considered significant at *p* < 0.05. SPSS version 16.0 (Chicago, IL, USA) was used for statistical analyses.

## 3. Results

Table 1 presents the demographic characteristics of the VG and the CG. The groups did not differ among the variables, including age, height, body weight, body mass index, and cognitive status (measured using the Mini-Mental State Examination). Participants reported no adverse effects from vibration.

After eight weeks of intervention, the results demonstrated that the muscle strength of the dominant and non-dominant wrist flexion (Z = −3.25, *p* = 0.001; Z = −2.06, *p* = 0.04), brachioradialis (Z = −2.43, *p* = 0.02; Z = −2.35, *p* = 0.02), biceps brachii (Z = −2.97, *p* = 0.003; Z = −2.65, *p* = 0.01), and non-dominant deltoid anterior (Z = −1.94, *p* = 0.04) in the VG improved significantly compared with that in the CG (Table 2). After intervention, patients in the VG had significantly increased IADLs (Z = −2.8, *p* = 0.01), ADLs (Z = −3.4, *p* = 0.001), muscle strength of dominant (Z = −3.4, *p* = 0.001) and non-dominant (Z = −3.2, *p* = 0.001) maximal grip strength, dominant (Z = −3.0, *p* = 0.002) and non-dominant (Z = −2.3, *p* = 0.03) wrist flexion, dominant (Z = −3.4, *p* = 0.001) and non-dominant (Z = −3.0, *p* = 0.002) wrist extension, and dominant (Z = −3.4, *p* = 0.001) and non-dominant (Z = 11.2, *p* = 0.001) brachioradialis in the forearms (Table 2). The results also showed that the VG had significantly improved muscle strength of the dominant (Z = −2.1, *p* = 0.04) and non-dominant (Z = −2.9, *p* = 0.003) biceps brachii, and dominant (Z = −3.4, *p* = 0.001) and non-dominant (Z = −2.4, *p* = 0.02) triceps brachii (Table 2). In addition, we also found that the muscle strength of the dominant (Z = −3.0, *p* = 0.003) and non-dominant (t = −2.7, *p* = 0.01) deltoid anterior, dominant (Z = −3.2, *p* = 0.001) deltoid lateral, dominant (Z = −2.5, *p* = 0.01) deltoid posterior, and both dominant (Z = −2.3, *p* = 0.02) and non-dominant (Z = −2.2, *p* = 0.03) supraspinatus in the shoulders increased in the VG (Table 2).

As shown in Table 3, an examination of the group × time interaction using GEE analyses revealed that only the muscle strength of the dominant (*ß* = 2.49, *p* = 0.002) and non-dominant (*ß* = 1.89, *p* = 0.02) wrist flexion, brachioradialis (*ß* = 3.8, *p* = 0.01), and biceps brachii (*ß* = 3.02, *p* = 0.02) in the dominant upper limbs were increased in the VG group compared to the CG (Table 3).

## 4. Discussion

To the best of our knowledge, this is the first study design to use a handheld vibrator with a specific focus on enhancing muscle strength in upper limbs among pre-frail older adults. The results demonstrate that muscle strength in the bilateral wrist flexors; dominant wrist extensors; bilateral brachioradialis; bilateral biceps brachii; dominant triceps brachii; and bilateral anterior, lateral, and posterior deltoid in the VG increased significantly after vibration exercises compared to the CG. These findings indicate that vibration exercises combined with multi-component training could enhance muscle strength in the upper limbs among pre-frail older adults.

### 4.1. Impacts of Combining Vibration and Multi-Component Exercises on Enhancing Muscle Strength and Functionality among Pre-Frail Older Adults

After eight weeks of intervention, the results showed an increase in muscle strength for the dominant brachioradialis and non-dominant biceps of 29.2% (*p* < 0.05) and 28.4% (*p* < 0.05), respectively, in the CG. In contrast, the VG showed increased muscle strength for the same muscles by 88.2% (*p* < 0.001) and 52.5% (*p* < 0.05), respectively. Meanwhile, the VG results also revealed significant muscle strength improvements for other muscles in the upper limbs, including the bilateral wrist flexion or extension; anterior, lateral, or posterior deltoid; and brachioradialis on the non-dominant side and the biceps in the dominant side by 20.7–65.9% (*p* < 0.05). Furthermore, the VG showed improved muscle strength for the bilateral wrist flexion, brachioradialis, and biceps between 35.6% and 54.8% (*p* < 0.05) compared to the CG.

These findings indicated that multi-component exercises could significantly improve muscle strength and that combining vibration and multi-component exercise results in better benefits on improving muscle strength in the upper limbs than the multi-component exercise programs only among pre-frail older adults. This aligns with the findings of previous studies. For example, a recent study reported that multicomponent exercise (including aerobic, resistance, and flexibility exercises) could improve the muscle strength and functions of frail and pre-frail older adults [41,42]. Silva et al. [25] also recruited healthy participants for an exercise combination program and asked them to sit with their elbows flexed at 90°, after which they were asked to perform exercises with vibrations applied (frequency: 8 Hz, amplitude: 6 mm) for 6 s, with 12 sessions, three sessions per week for four weeks. The results showed increased muscle strength by 26% in the upper limbs compared to the exercise program alone [25]. In addition, a recent meta-analysis demonstrated that whole-body vibration has comparable effects to resistance training for improving muscle strength [43]. However, another recent meta-analysis suggested that vibration was not effective in improving muscle strength among older adults with sarcopenia [44]. Recent studies have also revealed that the vibration frequency between 25–46 Hz can increase muscle activation and strength in the upper limbs [23,24,26]. In this study, we selected a 30 Hz vibration frequency and demonstrated improvements in muscle strength. These findings indicated that the different vibration approaches and relevant force transmission may affect muscle activation in the upper limbs and should be considered. Furthermore, the results of this study failed to show improvement in grip strength, ADL, and IADL scores in both the CG and VG, which could be because the participants were in the pre-frailty stage and showed partial exhaustion and low energy expenditure rather than the experienced degeneration in grip strength and functional limitations.

### 4.2. Different Vibration Approaches may Induce Different Force Transmission in Facilitating Muscle Activation in the Upper Limbs

In this study, we asked the participants to perform the vibration approaches by actively holding the vibrators and against the vibration force from the vibrator to maintain the training position. The results also indicated the benefits of enhancing upper-limb muscle strength among pre-frail older adults after eight weeks of vibration exercise. These positive findings are similar to previous studies that used whole-body vibration to induce muscle strength in the upper limbs, but more precisely, to facilitate muscle strength in the upper limb without inducing any inconvenience for participants. For example, recent studies have used the whole body vibratory platforms as the vibrator and recruited healthy participants, participants with spinal cord injuries or stroke patients to sit on a chair or kneel on the ground, put their upper limbs on the vibratory platform with hands or elbows [12,13,14,15,16,17,18,19], hold the handrail with both hands on the top of the vibratory platform [45], or sit on the platform directly [30]. Meanwhile, the whole-body vibratory approaches could deliver inappropriate and excess vibration to the head force from the whole-body vibration platform and result in vertigo, muscle soreness, and related discomfort [20,21]. In this study, we found no vertigo, pain, or discomfort among the participants during or after the vibration exercise, suggesting that the developed handheld vibrator may be better at improving muscle strength than the whole-body vibration. This may be because the muscles in the forearm and upper arm absorbed the vibration force while performing the vibration exercise, and few vibration forces were transmitted to the shoulders and trunk. Furthermore, these findings may indicate that vibration force transmission could decrease and result in lower muscle activation in the shoulder girdle because, in this study, muscle strength improvement was found to be greater in the forearm (88.2%) and less in the upper arm (56.6%) as well as the shoulder girdle (40.3%). However, this attribute could result from many factors and requires further investigation in future research. Furthermore, many studies also reported that a vibration intervention showed no significant improvement in muscle strength in upper limbs [46,47]. The different findings could result from the participants actively or passively performing the vibration programs [43,44]. In this study, we asked the participants in the VG to maintain the training position during the vibration intervention actively, so the participants had to generate more muscle strength against the vibration force from the vibrator to maintain the training position, resulting in further muscle activation and participation. Hence, the active vibration approach could be considered a complement to improve muscle strength for pre-frail older adults. Moreover, in the VG, the muscle strength of the supraspinatus in the bilateral upper limbs did not significantly improve after eight weeks of vibration exercise. However, the vibration force transmission and the relevant muscle activation in the upper limbs are complex processes influenced by biomechanics [45] and different vibration protocol parameters (frequency, amplitude, displacement, vibration time, types of vibration, and postures) [48]. Future studies must further investigate this aspect.

### 4.3. Study Limitations

Although this study demonstrated the positive effects of vibration exercises on enhancing muscle strength in the upper limbs among pre-frail older adults, it has some limitations. This study only indicated the positive effects of vibration exercises through a fixed 30-Hz vibration frequency and a 5-mm vibration amplitude. Therefore, the impact of different or progressive vibration protocol parameter settings is required in further studies. Although many studies have used a complete body vibration platform as the vibrator to enhance upper-limb muscle strength, we did not use this in the VG because of the potential risks (vertigo, muscle soreness, jaw, or neck discomfort) that may be induced [10,20,21]. Meanwhile, the different multi-component exercise programs for each group and the lack of previous and current history of exercise are also limitations in this study. Moreover, the results indicated that ADL and IADL might not be appropriate measurement tools for validating functional changes among frail older adults as they have a ceiling effect. Most participants scored 95–100 on the baseline assessment.

## 5. Conclusions

This study indicated that the eight-week combining vibration and multi-component exercise intervention showed more benefits in enhancing muscle strength than solely exercising in the upper limbs among pre-frail older adults. Hence, the vibration intervention can be a supplementary approach to enhance muscle strength in the upper limbs among pre-frail older adults.

## Figures and Tables

**Table 1:** Comparisons of Demographic Characteristics Between Groups.

Variable	All	CG	VG	*Z*	*p*
	*n* = 31	*n* = 16	*n* = 15		
Age (year)	75.4 (5.5)	74.9 (5.9)	75.9 (5.2)	−0.60	0.55
Body height (cm)	153.7 (4.7)	153.4 (4.2)	154.1 (5.4)	−0.35	0.73
Body weight (kg)	60.2 (7.3)	60.2 (6.8)	60.1 (8.0)	−0.26	0.80
Body mass index (kg/m^2^)	25.6 (3.5)	25.7 (3.2)	25.5 (3.9)	−0.10	0.92
MMSE	27.5 (2.1)	28.0 (1.9)	27.0 (2.3)	−1.25	0.21

Note. Data are presented as mean (SD); CG: control group; VG: vibration group; MMSE: Mini-Mental State Examination; *p* values are from Mann—Whitney U test.

**Table 2:** Comparisons of Outcome Indicators Between Baseline and Post-Intervention in the Control and Vibration Groups.

Variable	CG (*n* =16)				VG (*n* = 15)				Between Group		
	Baseline	After	*Z*	^a^ *p*	Baseline	After	*Z*	^a^ *p*	Baseline	After	
	Mean (SD)	Mean (SD)			Mean (SD)	Mean (SD)			*d*	*d*	^b^ *p*
IADL	21.4 (2.4)	20.8 (4.9)	−3.2	0.002	21.3 (2.4)	22.3 (1.6)	−2.8	0.01	−0.01	−0.22	0.55
ADL	98.8 (2.9)	97.5 (8.8)	−3.5	<0.001	100 (0)	100 (0)	−3.4	0.001	−0.62	−0.50	0.16
Maximal grip strength											
Dominant	15.3 (5.1)	18.4 (4.1)	−0.6	0.54	18.9 (4.9)	20.2 (4.1)	−3.4	0.001	−0.01	−0.46	0.21
Non-dominant	16.6 (5.0)	16.3 (4.0)	−0.2	0.84	16.5 (6.1)	18.2 (5.1)	−3.2	0.001	−0.21	−0.60	0.10
Wrist flexion											
Dominant	4.5 (1.8)	5.0 (1.7)	−3.2	0.001	4.4 (1.2)	7.3 (1.7)	−3.0	0.002	−0.12	−1.17	0.001
Non-dominant	4.7 (1.8)	5.0 (1.8)	−2.8	0.01	4.2 (1.0)	6.5 (1.6)	−2.3	0.03	−0.02	−0.74	0.04
Wrist extension											
Dominant	5.1 (1.9)	6.2 (1.5)	−2.8	0.01	4.8 (1.5)	6.5 (1.4)	−3.4	0.001	−0.12	−0.31	0.39
Non-dominant	4.6 (1.8)	5.5 (1.5)	−1.1	0.29	4.7 (1.6)	5.6 (1.8)	−3.0	0.002	−0.16	−0.02	0.95
Brachioradialis											
Dominant	7.2 (2.1)	9.3 (3.1)	−2.9	0.003	6.8 (1.6)	12.8 (3.9)	−3.4	0.001	−0.20	−0.87	0.02
Non-dominant	6.9 (2.7)	8.6 (3.2)	−0.3	0.78	7.3 (2.0)	11.9 (4.9)	−3.4	0.001	−0.29	−0.84	0.02
Biceps brachii											
Dominant	7.6 (1.7)	9.2 (2.7)	−3.3	0.001	8.3 (1.9)	13.0 (3.7)	−2.1	0.04	−0.46	−1.07	0.003
Non-dominant	6.7 (2.4)	8.6 (2.5)	−2.9	0.003	8.0 (2.1)	12.2 (4.1)	−2.9	0.003	−0.60	−0.95	0.01
Triceps brachii											
Dominant	8.6 (2.5)	10.5 (2.3)	−3.2	0.002	8.2 (1.5)	9.9 (1.7)	−3.4	0.001	−0.12	−0.17	0.65
Non-dominant	8.2 (2.6)	9.9 (2.4)	−0.6	0.57	8.7 (1.9)	9.6 (2.2)	−2.4	0.02	−0.31	−0.01	0.97
Deltoid anterior											
Dominant	7.0 (2.2)	8.2 (2.2)	−2.7	0.01	7.3 (1.9)	9.6 (1.8)	−3.0	0.003	−0.23	−0.63	0.08
Non-dominant	6.1 (2.6)	7.2 (2.3)	−1.9	0.05	6.4 (1.4)	8.9 (2.2)	−2.7	0.01	−0.41	−0.70	0.04
Deltoid lateral											
Dominant	7.0 (2.0)	7.9 (2.8)	−2.1	0.04	6.7 (1.9)	9.4 (2.5)	−3.2	0.001	−0.01	−0.62	0.09
Non-dominant	6.0 (2.6)	7.5 (2.7)	−1.1	0.26	6.3 (1.8)	8.5 (2.3)	−1.8	0.08	−0.47	−0.44	0.22
Deltoid posterior											
Dominant	6.5 (2.7)	8.2 (1.8)	−2.0	0.04	6.9 (2.3)	8.8 (2.2)	−2.5	0.01	−0.14	−0.25	0.49
Non-dominant	6.8 (3.0)	7.7 (2.0)	−0.9	0.38	6.9 (2.0)	8.8 (2.3)	−1.3	0.19	−0.09	−0.47	0.19
Supraspinatus											
Dominant	8.2 (1.4)	8.7 (2.5)	−1.0	0.33	7.7 (1.6)	8.6 (2.2)	−2.3	0.02	−0.42	−0.08	0.83
Non-dominant	7.6 (2.1)	8.0 (2.1)	−1.2	0.25	7.2 (1.7)	8.1 (2.0)	−2.2	0.03	−0.11	−0.14	0.71

Note. Data are presented as mean (SD); *p* values were from independent *t* test; CG: control group; VG: vibration group; ADL: activities of daily living; IADL: instrumental activities of daily living. ^a^ *p* values are from Wilcoxon Signed Ranks Test; ^b^ *p* values are from Mann—Whitney U test; *d*: standardized mean-difference effect size.

**Table 3:** Evaluation of the Intervention on Outcome Indicators Based on GEE Analysis.

Variable	Within-Time		Between-Group		Interaction Group (CG) × Time		
	Ref: Baseline		CG vs.VG		Ref: (VG) × Time		
	*ß*	*p*	*ß*	*p*	*ß*	95% CI	*p*
IADL	1.07	0.14	1.80	0.39	−1.69	−4.7−1.3	0.26
ADL	0.00	0.07	0.00	1.00	−1.25	−5.6−3.1	0.58
Maximal grip strength							
Dominant	1.28	0.43	−0.17	0.97	−0.85	−5.3−3.6	0.71
Non-dominant	1.74	0.38	−0.39	0.93	−0.78	−5.7−4.2	0.76
Wrist flexion							
Dominant	2.94	<0.001	2.65	0.03	−2.49	−4.0–−0.9	0.002
Non-dominant	2.23	<0.001	2.33	0.05	−1.89	−3.4–−0.4	0.02
Wrist extension							
Dominant	1.67	0.001	0.87	0.51	−0.60	−2.1−0.9	0.45
Non-dominant	0.92	0.13	−0.15	0.91	0.01	−1.6−1.6	0.99
Brachioradialis							
Dominant	5.91	<0.001	4.16	0.02	−3.80	−6.5–−1.1	0.01
Non-dominant	4.61	0.001	2.53	0.25	−2.93	−6.2−0.3	0.08
Biceps brachii							
Dominant	4.68	<0.001	2.27	0.18	−3.02	−5.6–−0.5	0.02
Non-dominant	4.19	<0.001	0.93	0.64	−2.24	−5.0−0.6	0.12
Triceps brachii							
Dominant	1.70	0.003	0.13	0.93	0.21	−1.7−2.2	0.83
Non-dominant	0.91	0.21	−1.23	0.49	0.73	−1.5−2.9	0.52
Deltoid anterior							
Dominant	2.32	<0.001	0.85	0.59	−1.11	−3.1−0.8	0.27
Non-dominant	2.56	<0.001	1.26	0.44	−1.52	−3.6−0.6	0.15
Deltoid lateral							
Dominant	2.69	0.001	1.98	0.23	−1.74	−4.0−0.5	0.13
Non-dominant	2.16	0.003	0.42	0.81	−0.72	−3.0−1.5	0.53
Deltoid posterior							
Dominant	1.95	0.02	−0.03	0.99	−0.30	−2.5−1.9	0.79
Non-dominant	2.03	0.01	1.11	0.56	−1.10	−3.4−1.2	0.34
Supraspinatus							
Dominant	0.92	0.17	0.92	0.49	−0.41	−2.3−1.5	0.67
Non-dominant	0.91	0.16	0.94	0.53	−0.51	−2.4−1.4	0.59

Note. ß: Regression coefficient; CI: confidence interval; GEE: generalized estimating equation; CG: control group; VG: vibration group; ADL: activities of daily living; IADL: instrumental activities of daily living. Models were performed using a GEE with a Group × Time interaction term characterizing the intervention effect of interest.

## Data Availability

Upon a reasonable request, all the data collected and analyzed throughout this study are available from the corresponding author.
